# Reliability-Based Weighting of Visual and Vestibular Cues in Displacement Estimation

**DOI:** 10.1371/journal.pone.0145015

**Published:** 2015-12-14

**Authors:** Arjan C. ter Horst, Mathieu Koppen, Luc P. J. Selen, W. Pieter Medendorp

**Affiliations:** Radboud University, Donders Insitute for Brain, Cognition and Behaviour, Nijmegen, The Netherlands; Centre de Neuroscience Cognitive, FRANCE

## Abstract

When navigating through the environment, our brain needs to infer how far we move and in which direction we are heading. In this estimation process, the brain may rely on multiple sensory modalities, including the visual and vestibular systems. Previous research has mainly focused on heading estimation, showing that sensory cues are combined by weighting them in proportion to their reliability, consistent with statistically optimal integration. But while heading estimation could improve with the ongoing motion, due to the constant flow of information, the estimate of how far we move requires the integration of sensory information across the whole displacement. In this study, we investigate whether the brain optimally combines visual and vestibular information during a displacement estimation task, even if their reliability varies from trial to trial. Participants were seated on a linear sled, immersed in a stereoscopic virtual reality environment. They were subjected to a passive linear motion involving visual and vestibular cues with different levels of visual coherence to change relative cue reliability and with cue discrepancies to test relative cue weighting. Participants performed a two-interval two-alternative forced-choice task, indicating which of two sequentially perceived displacements was larger. Our results show that humans adapt their weighting of visual and vestibular information from trial to trial in proportion to their reliability. These results provide evidence that humans optimally integrate visual and vestibular information in order to estimate their body displacement.

## Introduction

To accurately navigate through the environment, we not only need to know the direction in which we move, called heading, but we also need to keep track of our displacement. In inferring these components of self-motion, the brain may rely on various sensory modalities, especially visual (e.g., optic flow) [1] and vestibular information [2–9].

Heading estimation has received much attention, particularly with regard to how the brain combines overlapping but noisy information from different modalities. In recent years this problem has typically been approached from a Bayesian inference perspective. In this framework an optimal observer reduces the uncertainty in the internal estimate of heading by weighting each signal in proportion to its reliability [3, 10–17]. This framework still holds when visual cue reliability varies unpredictably across trials. This notion of dynamic cue reweighting in multisensory heading perception has been validated in recent years, both at the behavioral [2] and the neural level [18].

Would the same computational principle also hold up fort the estimation of travelled distance? For several reasons, displacement estimation may not rely on the same neural computations as heading estimation. First, heading estimation can be derived from the instantaneous direction of translation and may improve through evidence accumulation with the ongoing motion [19]. The estimate of displacement, in contrast, requires the combining of sensory information and (double) integration of the acceleration and velocity information across the whole displacement, i.e., from start to end of the motion. Second, geometrically, while optic flow signals can readily be used to estimate heading [1], they require depth scaling to provide unambiguous cues to travelled distance [20]. Third, heading and displacement detection thresholds appear to be susceptible to motion directions in different reference frames [9].

Thus far, most studies on displacement estimation have been limited to testing the contribution of single cues, such as vision [20–23] or vestibular input [6, 8, 24]. Only a few studies reported on the interplay between visual and vestibular information during displacement estimation. In the study by Harris et al. (2000) subjects had to indicate the position of a previously perceived visual or physical stop target by a button-press when “passing” that target during passive translation with either ‘only visual’, ‘only vestibular’ or ‘visual and vestibular’ information being present [4]. They observed differences in the accuracies when comparing displacement estimates from different modalities, i.e., visual or vestibular. Another study tested absolute displacement estimation in an oscillatory up-down movement [5]. They showed that a model of gain-dependent linear weighting of visual and vestibular cues could explain their results for in-phase, but not out-of-phase, visual and vestibular motion. Along the same line, Campos et al. (2012) used a task in which subjects had to match a previous displacement by adjusting the distance of a virtual target [25]. Using the accuracies of displacement estimates in single and combined cue conditions, they were able to predict the observed linear weighting of discrepant visual and vestibular cues. The latter two studies [5, 25]explained their data using a linear integration model. However, because they did not experimentally vary cue reliability, it is unknown whether this integration was performed optimally by combining displacement cues relative to their reliability. Therefore, the objective of the present study is to examine whether visual and vestibular cues are dynamically weighted in displacement estimation when cue reliability is varied randomly across trials.

Here, using a psychometric approach, we tested whether observers show optimal visual and vestibular cue integration in displacement estimation for different levels of cue reliability, analogous to previous experiments on heading estimation in humans and macaque monkeys[2]. By manipulating cue reliability randomly across trials, we were able to test whether cue weights are dynamically updated at the single trial level. We found that human subjects optimally integrate visual and vestibular cues by dynamically adjusting cue weights depending on their relative reliability from trial to trial.

## Materials and Methods

### Participants

Six healthy subjects (4 female), 19–25 years old participated in the study. All subjects had normal or corrected to normal vision, including normal stereovision (tested using the Randot Stereo test (Stereo Optical Inc., Chicago, USA)) and no known history of neurological, visual, or vestibular sensory disorders. Informed written consent was obtained from all subjects prior to the experiment and the experiment was approved by the Ethics Committee Faculty of Social Sciences (ECSS).

### Equipment

**Fig 1:**
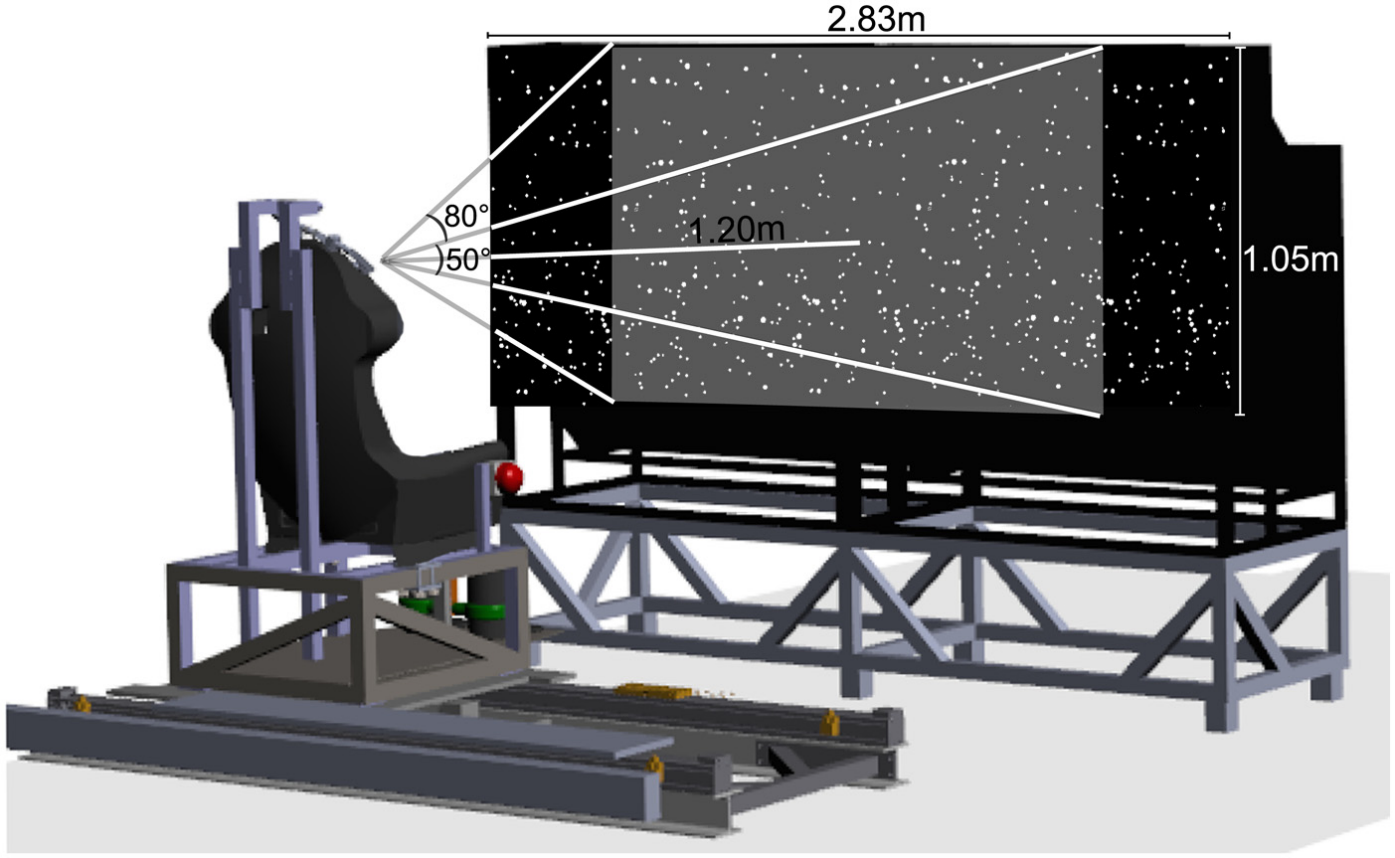
Schematic view of the setup. Left the movable chair on a rail and at the right the 3D screen with a cloud of stars.

Vestibular stimuli were administered using a linear sled on an 800-mm track with which subjects were laterally translated. The sled, powered by a linear motor (TB15N, Technotion, Almelo, The Netherlands), was controlled by a Kollmorgen S700 (Danaher, Washington, DC) drive. The kinematics of the trajectory of the subjects were accurately controlled at 1000 Hz with accuracy better than 34 μm, 2 mm/s, and 150 mm/s^2^. Subjects were seated on the sled with their interaural axis aligned with the sled’s motion axis and were restrained using a five point seat belt and a chin rest (Fig 1). The head was firmly held in place using a chin rest and an ear-fixed mold. Cushioning was provided at the back and sides of the head for comfort and to prevent confounding cues related motion related vibrations. White noise was administered through headphones to prevent the subjects from using motion related noise of the sled to be used as a cue.

Visual stimuli were projected using two digital stereo DLP^®^-rear projection cubes (EC-3D-67-SXT+ -CP, Eyevis GmbH, Reutlingen, Germany) on a 2.83 X 1.05 m surface with a resolution of 2800 by 1050 pixels. Subjects were seated 1.2 m in front of the screens, which thus subtended 97.3° X 45.4° of visual angle. Vertical retraces of the screens were synchronized using an Nvidea Quadro K5000 graphics card. The visual display was updated at 60 Hz. Stereoscopic images were generated using channel separation, based on interference filter technology (INFITEC^®^ GmbH, Ulm, Germany). Images for the left and right eye were projected at different wavelengths. Subjects wore a pair of glasses with selective interference filters for each eye. Visual stimuli depicted optic flow that accurately simulated movement of the observer through a world stable cloud of random stars in a virtual space of 3m wide, 2m high and 1.2m deep (0.6 m in front and 0.6 m behind the screen). Star density was 0.01/cm³, with each star being a 0.15 cm X 0.15 cm triangle. The visual stimulus also contained a white head-fixed fixation point at screen depth at which the subjects had to fixate during the trial. The visual scene provided multiple depth cues such as relative size, motion parallax and binocular disparity.

Projection of perspective changes due to motion was controlled by custom developed python software, using OpenGL. The sled position was sampled at 1000Hz and by using an extrapolation paradigm on the current and previous sled positions we predicted the position on t + 66.67ms to overcome the latency of the projection system. This predicted position was used as observer position in our OpenGL software to render the 3D environment. In the case of only visual cues and no self-displacement, a sled-movement was simulated to administer the same visual cues to the observer as during a condition in which visual and vestibular (i.e., self-motion) cues were presented.

### Procedure

The task consisted of a two-interval two-alternative forced-choice (2AFC) task in which subjects had to judge whether the second of two consecutive movements was shorter or longer than the first. One of the intervals contained the “reference” movement the other imposed the “probe” movement. The reference movement consisted of a rightward translation of 20 cm and the probe movement was also rightward but varied in fine steps around the reference amplitude using the adaptive Ψ-method [26], but was limited between 5cm and 35 cm. The order of reference and probe movement was counterbalanced across trials. Subjects were unaware of the order of the reference and probe movements. At the end of the second movement subjects were presented an auditory cue indicating they could respond. After the response was given, subjects were translated leftward to the starting position. In each trial subjects were presented with two 1.5s translational motion stimuli with a bell-shaped velocity profile (peak velocity = 0.25 m/s, peak acceleration = 0.98 m/s² for the reference movement). Both motion profiles were delivered in one of three randomly interleaved stimulus modalities: vestibular only (inertial movement without optic flow in complete darkness), visual only (optic flow without inertial movement) and combined (synchronous inertial movement and optic flow). Within a trial the motion profiles only differed in amplitude and the stimulus properties remained the same (modality and coherence). We note that during inertial movement, also extra-vestibular cues were available to the subjects such as somatosensation and proprioception; however, we refer to this condition as vestibular because both behavioral and electrophysiological performance has been shown to strongly depend on intact vestibular labyrinths [16].

**Fig 2:**
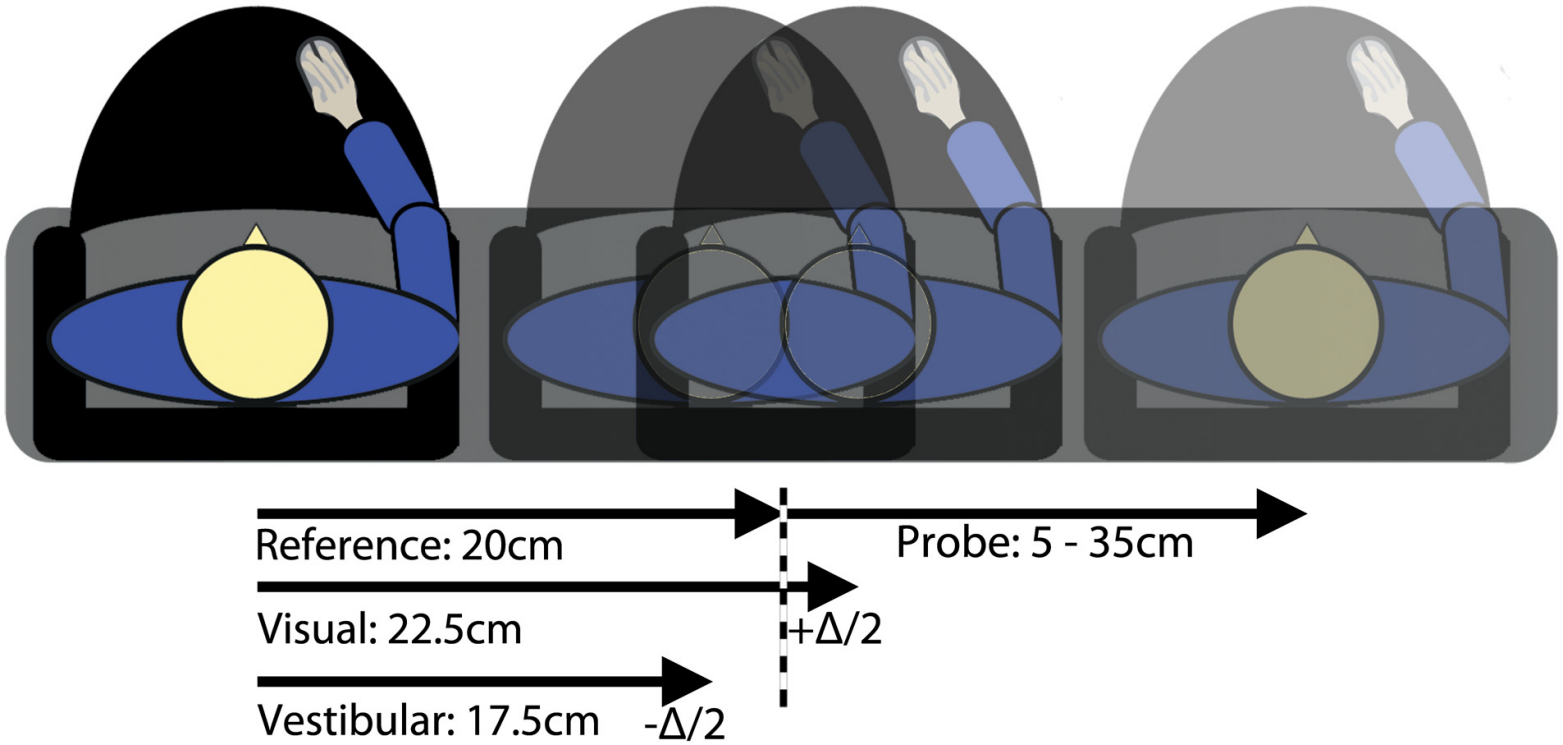
Schematic representation of the displacement estimation task. Shown is a combined condition with visual-vestibular discrepancy. Reference motion was always 20cm +Δ, with Δ = -5, Δ = 0 or Δ = +5 cm, depending on the condition. Probe motion was between 5 and 35cm. Probe amplitude was determined using the adaptive Psi-procedure.

Combined condition trials were randomly assigned one of three conflict amplitudes: +Δ,—Δ, or 0 (no conflict), with Δ being 5cm. Conflict amplitudes only existed in the reference movement (Fig 2). Positive Δ indicates smaller movement amplitude for vestibular relative to visual cues (vice versa for negative Δ). When Δ was nonzero, the vestibular displacement was 17.5 cm or 22.5 cm and visual displacement was 22.5 cm or 17.5 cm for Δ = 5 cm and Δ = -5 cm, respectively.

During the experiment, visual cue reliability was manipulated by changing the motion coherence of the optic flow pattern by randomly replacing a fixed percentage of the stars in each frame. This visual motion coherence was manipulated in three steps, i.e. 20%, 60% and 100%, randomly applied across trials. Vestibular cue reliability was held constant. All stimulus triangles had a limited lifetime of 30 frames i.e., approximately 500ms. Prior to the movement the stars were randomly divided into 30 triangle-sets. Every frame one of these sets was re-initialized at a new random location in the world and this location remained stationary for the next 30 frames, i.e. was drawn in accordance with the observers’ displacement.

Each subject was presented with 13 different conditions, see Table 1, divided over 5 sessions of one hour each. All visual only conditions were randomly interleaved as well as the combined conditions, though both sets of conditions were acquired separately. Also the vestibular only condition was acquired in a separate session. Prior to each set of conditions, subjects conducted 25 practice trials leading to a total of 2025 trials (13 conditions * 150 trials + 3 practice blocks * 25 trials).

**Table 1:** Conditions with corresponding cues, motion coherence and discrepancy.

Condition	Cue	Motion Coherence (%)	Δ (cm)
**1**	Vestibular	NA	NA
**2**	Visual	100	NA
**3**	Visual	60	NA
**4**	Visual	20	NA
**5**	Combined	100	-5
**6**	Combined	60	-5
**7**	Combined	20	-5
**8**	Combined	100	0
**9**	Combined	60	0
**10**	Combined	20	0
**11**	Combined	100	+5
**12**	Combined	60	+5
**13**	Combined	20	+5

### Theory and model predictions

Given the visual *S*_*vis*_and vestibular *S*_*ves*_ sensory estimates of an environmental property*S*, the joint likelihood of these estimates can be described as *P*(*S*|*S*_*vis*_,*S*_*ves*_). Using Bayes’ rule, this probability can be re-written as

$$P\left(S|S_{vis},S_{ves}\right)\propto P\left(S_{vis},S_{ves}|S\right)P\left(S\right)$$ (1)

Assuming a uniform prior and independent noise for the single cues, Eq 1 can be rewritten as

$$P\left(S|S_{vis},S_{ves}\right)\propto P\left(S_{vis}|S\right)P\left(S_{ves}|S\right)$$ (2)

When the likelihood functions of the two single cues are considered to be Gaussian, the posterior probability will also be Gaussian. This results in a simple statistical optimal estimation model in which the means ${\hat{S}}_{ves}$ and ${\hat{S}}_{vis}$, and variances ${\hat{\sigma }}_{ves}^{2}$ and ${\hat{\sigma }}_{vis}^{2}$ of the single cue distributions can be used to obtain the combined mean ${\hat{S}}_{comb}$ and variance ${\hat{\sigma }}_{comb}^{2}$. We find:

$${\hat{S}}_{comb}={\hat{w}}_{vis}{\hat{S}}_{vis}+{\hat{w}}_{ves}{\hat{S}}_{ves},$$ (3)

$${\overset{\wedge }{\sigma }}_{comb}^{2}=\frac{\left({\overset{\wedge }{\sigma }}_{{}_{vis}}^{2}{\overset{\wedge }{\sigma }}_{ves}^{2}\right)}{\left({\overset{\wedge }{\sigma }}_{{}_{vis}}^{2}+{\overset{\wedge }{\sigma }}_{ves}^{2}\right)},$$ (4)

with

w^ves=1σ^ves21σ^vis2+1σ^ves2, (5)

w^vis=1σ^vis21σ^vis2+1σ^ves2.

Thus, in this optimal integration model the combined mean is the weighted average of the individual mean estimates, with weights proportional to the reliability (i.e. the inverse of the variance) of the corresponding cues, and the estimated combined variance is smaller than either of the unimodal variances, with the largest improvement (by a factor of 2) when the single cue variances are equal.

The predicted combined cue variance and optimal weighting for the combined mean based on the individual cues’ variances are compared to the observed combined cue variance and the observed weights in the data. Assuming that both the vestibular and visual sensors are accurate, experimental information about the weighting of the single cues is only available in the combined conditions that have a discrepancy between the visual and vestibular cues, i.e. the Δ = -5 and Δ = +5 conditions [2]. We used these data as follows. Consider the case of positive Δ, i.e., with the visual cue amplitude smaller than the vestibular cue. The weighting can be estimated by measuring the shift of the point of subjective equality (PSE) relative to the zero conflict combined condition. When the PSE is shifted by +Δ/2, the subject estimated 50% of the trials in which the probe was as large as the vestibular reference cue (cue conflict was only applied to the reference) to be larger than the reference. This can only be the case when the subject completely relies upon the vestibular cue, i.e., with a vestibular weight of 1. Following this analysis, the observed weights were estimated by rewriting Eq 3, again assuming accurate sensors:

$$\mu_{Comb}=\omega_{Vis}\mu_{Vis}+\left(1-\omega_{Vis}\right)\mu_{Ves}$$

$$\omega_{Vis}=\frac{\mu_{Comb}-\mu_{Ves}}{\mu_{Vis}-\mu_{Ves}}$$

Substituting the discrepancy terms (-Δ/2 and +Δ/2 for vestibular and visual cues, respectively) we obtain:

$$\omega_{Vis}=\frac{\mu_{Comb}+\frac{\Delta }{2}}{\frac{\Delta }{2}+\frac{\Delta }{2}}$$

resulting in:

$$\omega_{Vis}\left(observed\right)=\frac{\mu_{Comb}+\frac{\Delta }{2}}{\Delta }$$

$$\omega_{Ves}=1-\omega_{Vis}$$ (6)

### Data analysis

Analyses were performed using Matlab R2012a (MathWorks) and SPSS Statistics 19 (IBM). Response data was summarized by fitting a cumulative Gaussian to the proportion of “probe amplitude larger than the reference” responses as function of probe amplitude. The psychometric threshold and point of subjective equality (PSE) were taken as the SD (σ) and mean (μ), respectively. Furthermore, the lapse rate (typically represented by λ) was set at 0.04. Psychometric curve parameters (μ, σ) were fitted for each subject, stimulus condition, visual coherence level, and discrepancy using Maximum Likelihood Estimation (MLE). These parameters were inserted into Eqs 4–6 to predict the combined thresholds and weights.

Optimal integration predicts the combined threshold to be smaller than that of either single cue condition, although the size of this effect will depend on the discrepancy between the two single cue thresholds. That is, with large differences between the single cues in the low coherence levels the combined threshold will not decrease much with respect to the lowest single cue threshold, see Eq 4. We performed a direct comparison between the lowest single cue and combined thresholds for each coherence level separately using single tailed paired t-tests. We also tested whether the predicted and observed thresholds differ with a two-tailed paired t-test for each coherence level. In addition, we tested the effect of adding a discrepancy versus no discrepancy by using two-tailed paired t-tests, one for each coherence level. To test the effect of coherence level on dynamic weighting we tested whether the predicted and observed weights differ in a 2x3 analysis of variance (ANOVA) with the following design: 2 levels of outcome (predicted, observed) and three levels of visual motion coherence level (20%, 60%, 100%). Furthermore, to provide insight in individual differences we compared observed and predicted response patterns per subject for the thresholds and weights. To this end we computed the 95% confidence intervals (CIs) for the unimodal and combined cue thresholds using a bootstrap procedure. We randomly sampled 150 data points per condition with replacement. We then calculated the predicted combined variance using Eq 4, the predicted single cue weight using Eq 5 and the observed single cue weights using Eq 6. By repeating this procedure 999 times we constructed the upper and lower bounds of the 95% CI of the mean. These 95% confidence intervals were used to detect individual straying from optimality.

## Results

**Fig 3:**
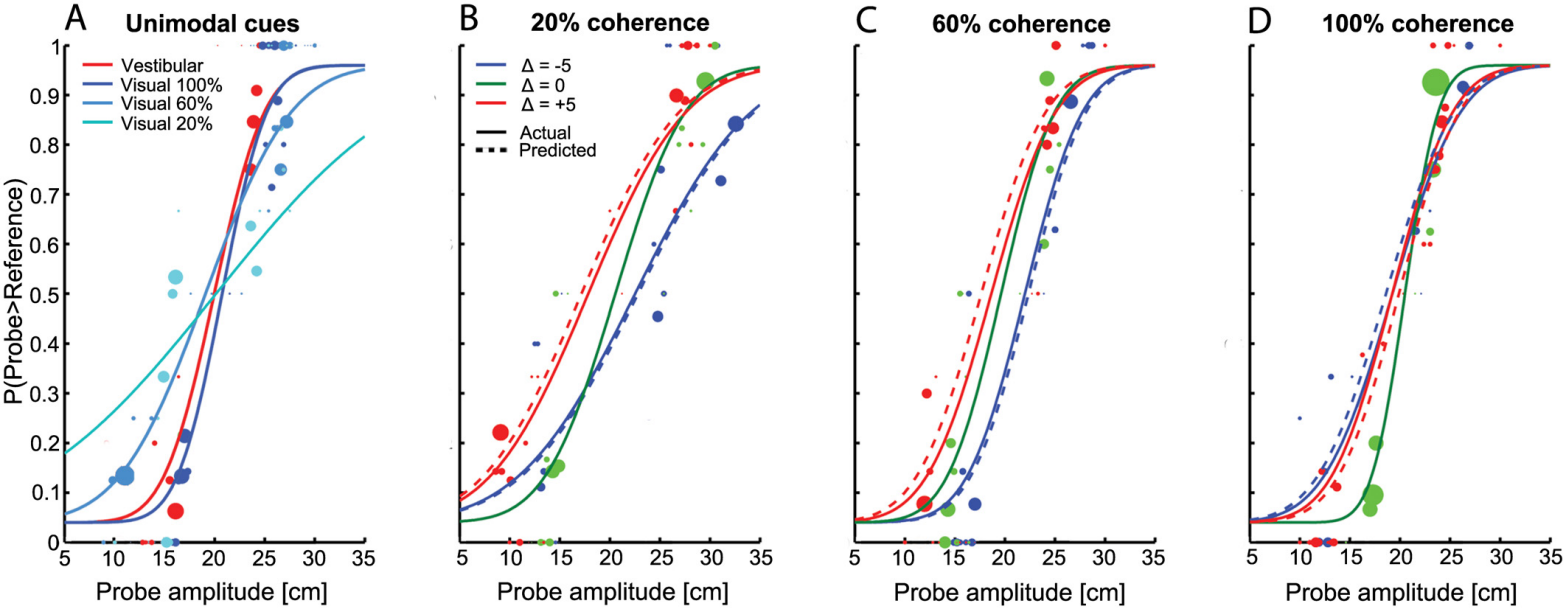
Example psychometric functions. Raw data (size of data points representing number of repetitions) is shown for one subject. Plotted is the proportion of probe larger than reference responses against probe amplitude. For single cue conditions (A), separate curves are plotted for the vestibular (red) and visual conditions with 100% (blue), 60% (light blue) and 20% (cyan) visual motion coherence. Combined data are represented in different plots (B-D) for the levels of motion coherence and in each plot data are distinguished by probe amplitude discrepancy [blue: Δ = -5 (visual < vestibular), green: Δ = 0 (cues consistent), red: Δ = +5 (visual > vestibular)]. Note that in (D) the discrepancy curves (i.e., with Δ = ±5 cm) differ only slightly from the non-discrepancy curve due to a 50% weighting of the visual cue for this particular subject. Dashed curves represent predicted psychometric functions for each Δ, based on the predicted cue weights (Eq 5).

Fig 3 shows the single and combined cue results of a typical subject. These psychometric curves illustrate the proportion of ‘larger’ responses as a function of probe amplitude. The different slopes of the psychometric functions in Fig 3A, quantified by their threshold, indicate that visual cue reliability depends on visual coherence levels. From the single cue thresholds, we predicted the weights that the subject should use when combining both cues in a statistically optimal manner. Each coherence level has different predicted weights, computed from each pairing of vestibular and visual single cue thresholds using Eq 5.

### Single cue and combined thresholds

Within Fig 3B–3D the predicted (dashed) and actual results are shown for all combined cue conditions; different panels are organized by the visual motion coherence levels. When visual cue reliability was low (i.e., coherence 20%, see Fig 3B), the combined cue psychometric functions during cue conflict (i.e., Δ = -5cm or Δ = +5cm) shifted according to vestibular dominance, that is, low visual weights (negatively biased red curves and positively biased blue curves). In contrast, when visual reliability was high (i.e., coherence 100%), the curves shifted based on visual dominance (i.e., higher visual weights), see Fig 3D. Overall, the observed results match the optimal predictions for all three coherence levels quite well.

**Fig 4:**
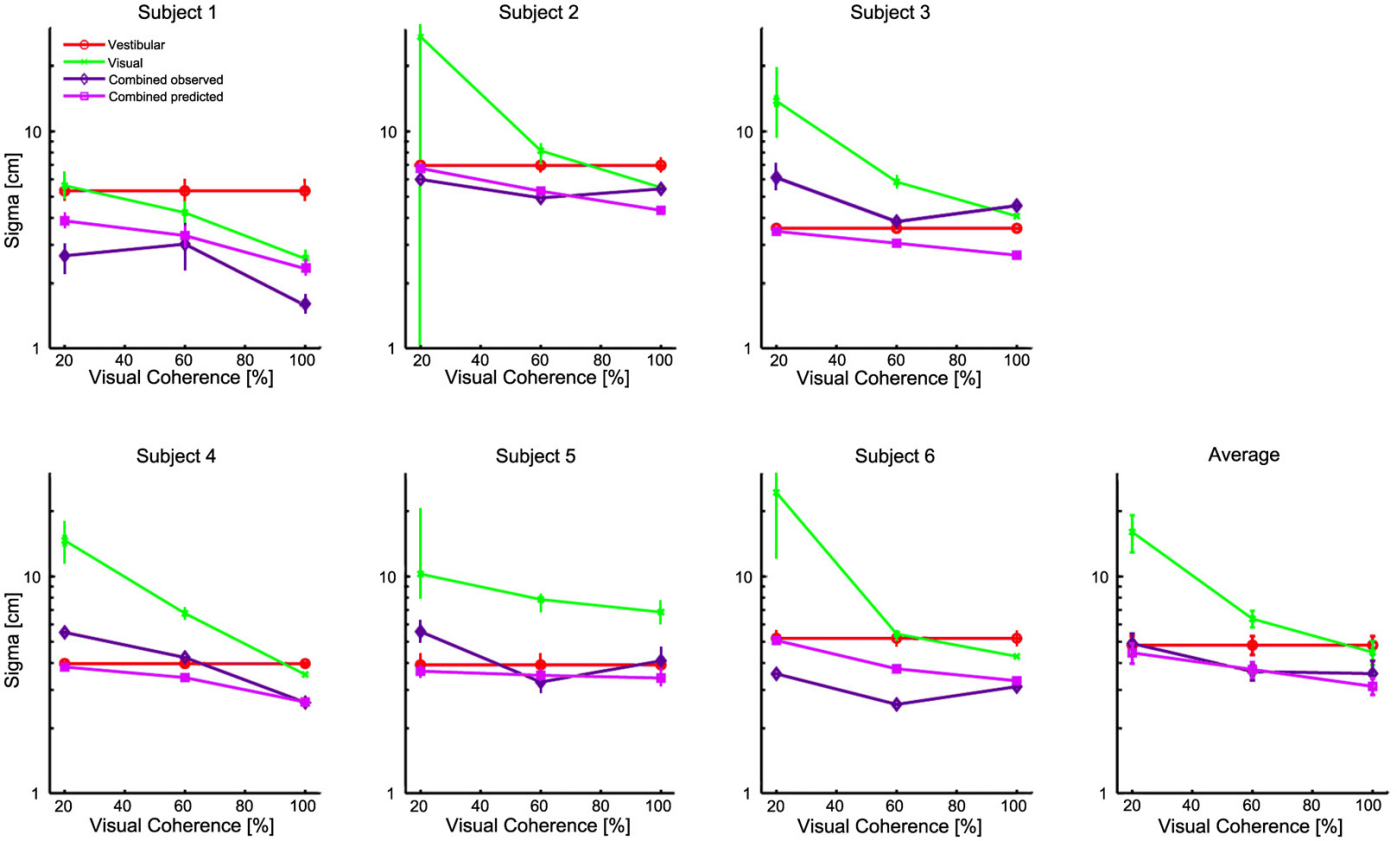
Psychophysical thresholds. Thresholds as function of visual motion coherence level for each subject (A-F) and their average (G). Single-cue vestibular (red) and visual (green) thresholds are shown together with the measured combined with Δ = 0 (purple) and predicted thresholds (magenta). Error bars represent the standard error of the mean (SEM). Error bars in A-F represent the Standard Error of the Mean (SEM) using the bootstrap procedure as explained in the Data-analysis section. Error bars in G represent the SEM across subjects. Vestibular thresholds, by definition, do not vary with coherence, but are the same data plotted at each point for comparison.

Fig 4A–4F shows the individual subjects’ actual and predicted thresholds for the single cue and combined conditions with Δ = 0. Fig 4G shows the average result across subjects. As mentioned, optimal cue integration models predicts the combined threshold to be lower than the lowest single cue threshold (Eq 4). We validated this prediction: we found a significant reduction in the combined threshold as compared to the lowest single cue threshold for 100% (t(5) = 2.23, *p* < 0.05) and 60% (t(5) = 2.68, *p* < 0.05), but not for 20% (*p* = 0.47) of visual motion coherence. In comparing the actual and predicted thresholds in the combined condition we found no significant differences (all *p* > 0.3). Notably, subject 2 shows large variability in the visual threshold in the 20% visual motion coherence condition. This can possible be explained by the large variability in the responses resulting in a relatively high visual threshold and a large variance in thresholds obtained via the bootstrap procedure.

We also tested the effect of whether adding a discrepancy on the combined cue thresholds. The results show a higher threshold for the nonzero- than for the zero delta conditions for 100% (t(5) = 2.673, *p* < 0.05) and 60% (t(5) = 6.208, *p* < 0.005) of visual motion coherence, but not for 20% (*p* = 0.12).

**Fig 5:**
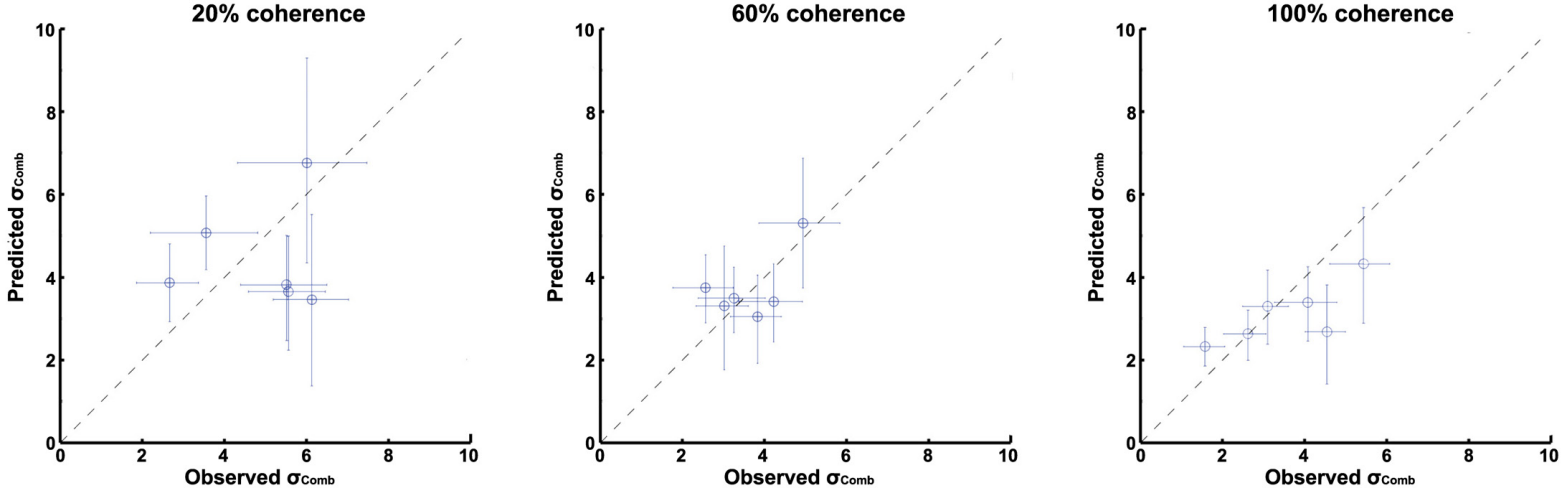
Scatterplot of observed and predicted combined cue thresholds. Different plots (A-C) represent different visual coherence levels, 20%, 60% and 100%, respectively. Error bars represent the 95% CI.

In Fig 5, individual differences between the predicted (Eq 4) and observed (Eq 6) combined cue thresholds can be observed. Shown are the estimates with their 95% confidence intervals (CI’s). As can be seen, the diagonal line, representing optimality, falls within CI’s of all but two subjects in the 60% and 100% coherence conditions. Interestingly, larger deviations from optimality can be observed for the 20% coherence condition, with the CI’s of 5 subjects falling outside the diagonal.

### Reliability based cue weighting

**Fig 6:**
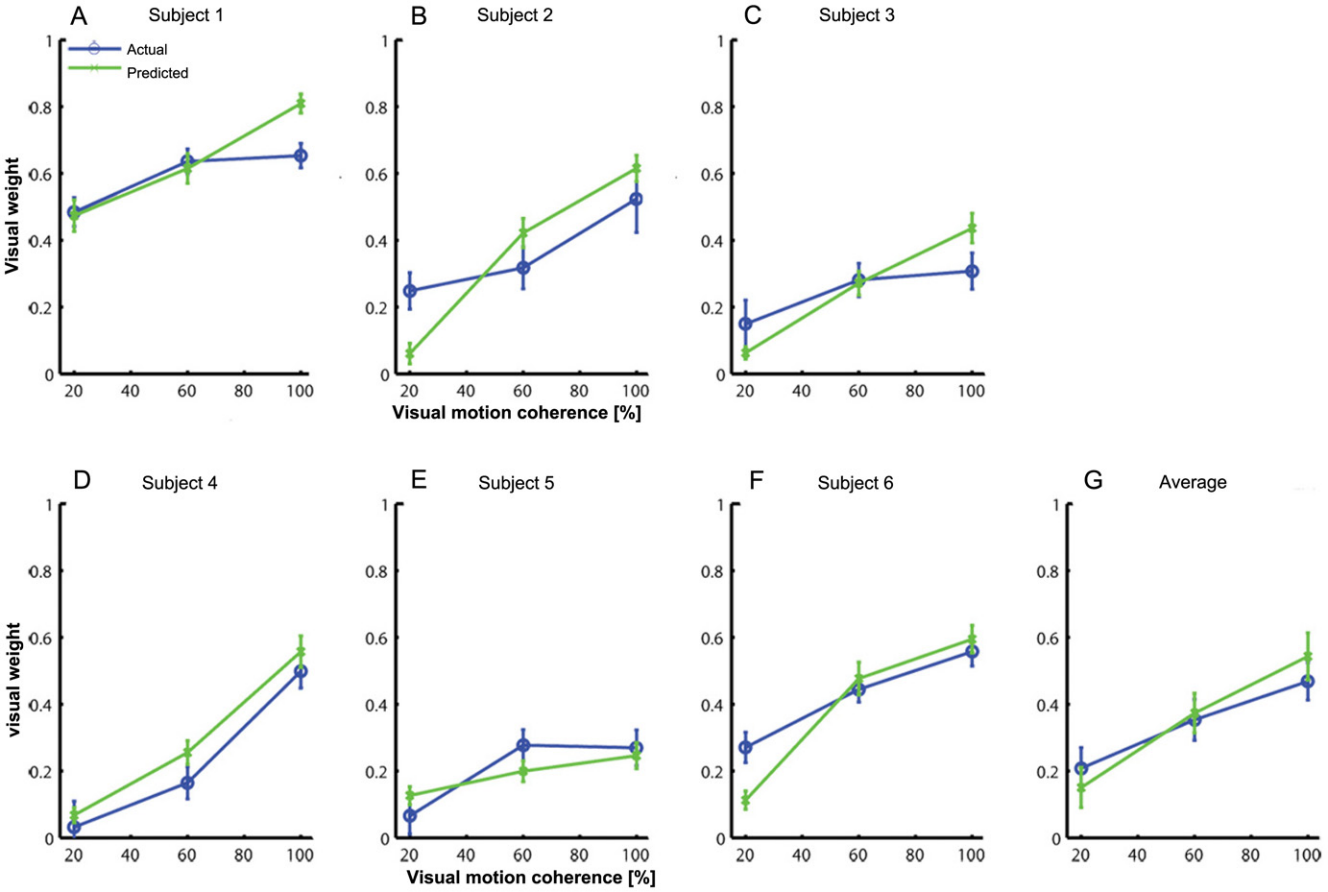
Visual weights as function of visual motion coherence level. Shown are visual weights. for each subject (A-F) and their average (G). Predicted (green) and observed weights (blue), according to eqs 5 and 6, respectively, are shown. Error bars in panels A-F represent the SEM based on a bootstrap procedure as explained in the Data-analysis section. Error bars in G represent the SEM across subjects.

Predicted and observed weights as obtained from Eqs 5 and 6, respectively, are summarized in Fig 6. The general result is that all subjects show robust changes both in actual and predicted weights as function of visual motion coherence. Statistical test results show no significant difference between predicted and observed visual weights (*p* > 0.41; η² = 0.136), or interaction with motion coherence (*p* > 0.08; η² = 0.397). The main effect of motion coherence was significant [F(2,10) = 35.406; *p* < 0.001; η² = 0.867] and reflects the reliability-dependent weighting, with higher visual weighting for reliable visual stimuli and lower visual weighting for less reliable visual cues.

**Fig 7:**
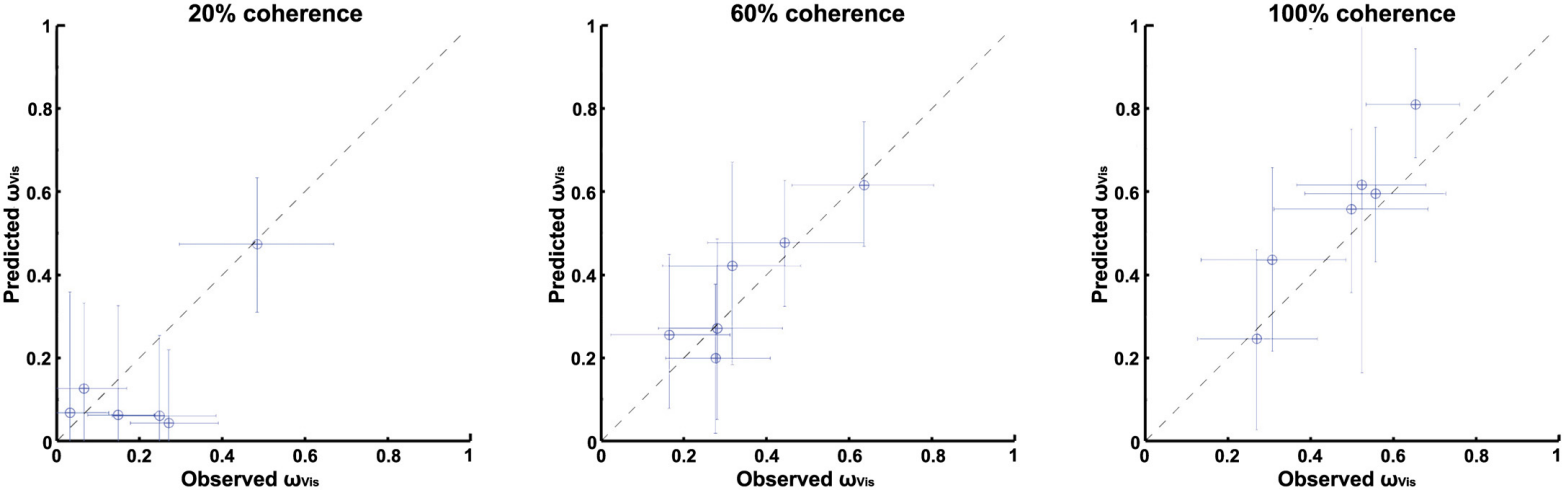
Scatterplot of observed and predicted visual weights. Plots A-C represent different visual coherence levels, 20%, 60% and 100%, respectively. Error bars represent the 95% CI.

In analogy to the individual threshold differences, individual differences in visual weighting can be observed, as thresholds and weights are closely related, see Eq 5. In order to gain more insight in these differences we plotted the observed and predicted weights in a scatterplot, as shown by Fig 7. For the 100% and 60% visual coherence conditions the observed and predicted visual weights match closely. In analogy to the combined threshold values, for the 20% coherence condition some deviations from optimality can be observed.

## Discussion

We studied the integration of visual and vestibular cues in a whole body lateral displacement discrimination task. Visual cues were presented with different levels of visual coherence (20%, 60% and 100%) to change relative cue reliability and discrepancies between visual and vestibular cues were imposed to test relative cue weighting. Our results are generally in line with two primary predictions of optimal integration schemes as derived from Bayesian inference theory: 1) human subjects reduce the variance of the combined cue percept and 2) they dynamically weight individual cues based on their reliabilities. Our results are therefore complementary to studies on multisensory integration during heading perception [2, 3, 11].

### Visual-vestibular interaction

Within the literature on the integration of visual and vestibular cues during self-motion a distinction can be made between studies addressing visual-vestibular integration from a *reliability-based* or *gain-based* cue combination perspective. Studies on reliability based cue weighting use probabilistic inference to determine the relative (weighted) contribution of single cues based on their reliability. In contrast, studies on gain-based cue weighting used differences in displacement estimates to determine cue weighting.

Several heading estimation studies showed cue reliability dependent weighting of cues (e.g., [2, 3]). Our study shows dynamic cue weighting for displacement estimation. The interaction of visual and vestibular cues during *heading* perception is consistently characterized by an overestimation of vestibular cues [2, 11, 13]. More specifically, the observed vestibular weighting is consistently higher than the predicted vestibular weighting based on single cue reliability. In contrast, our study does not show a significant vestibular overestimation. This overrating during heading estimation has been attributed to the use of virtual reality systems [13] or explained as the result of training effects [2]. Another, but yet to be explored possibility is the involvement of causal inference, in which multisensory integration is performed in two steps [27]. The first step determines the information provided by each cue and in the second step the likelihood of two cues coming from the same source or from multiple sources is estimated. When visual and vestibular displacement cues provide conflicting information, causal inference decides whether the displacement was indicated by a single cue or both cues. In the case of too large conflicts this may lead to the observer using only one cue to perform the task. Although the involvement of causal inference has been proposed as a possible explanation for the vestibular overweighting during heading discrimination [2, 13], it is still a speculative account [28]. As our results show no differences between the predicted and observed vestibular weights, we have no reason to assume that causal inference would have resulted in cue segregation.

Studies on *gain based* cue integration show controversy with regard to the relative weighting of visual and vestibular cues. In an experiment in which subjects had to match a predefined walked distance by adjusting a target in the depth dimension [25] showed nearly equal weighting of visual and vestibular cues, 0.54 and 0.46, respectively. Similarly, [5] showed nearly equal weighting when asking for absolute displacement estimates in an oscillatory up-down movement with 0.35–0.42 and 0.58–0.65 for the visual and vestibular weights, respectively, depending on motion amplitude. Despite the fact that our study uses a reliability based approach for the weighting of visual and vestibular cues, our results show remarkable agreement with the findings by [25] and [5]. They exhibit, on average, a nearly equal weighting for the single cues (visual: 0.46, vestibular: 0.54) with 100% visual coherence. In contrast, the study by [4] reports a dominance of non-visual cues in displacement estimation. They used a fore-after movement and had people indicate the position of a previously perceived visual or physical stop target by a button-press when “passing” that target during passive motion. There are several possible reasons for the inconsistency between the results of [4] and those of the current study. First, it has been argued that the relative importance of visual and vestibular cues is task dependent, with neither of the cues covering all aspects of self-motion perception [29]. Differences might already occur at the sensory level as vestibular detection thresholds and neural firing patterns of primary otolith afferents differ between motion directions [30, 31]. Second, in the study by [4] the visual stimulus was presented without stereoscopic vision. It is believed that adding stereoscopic vision facilitates the integration of visual and vestibular cues by disambiguating depth percept (but see [2, 12]). Third, the distances used by [4] are many times larger (1.5 m up to 10 m) than those used by us (0.05 m up to 0.35 m). According to Weber’s Law, increases in discrimination thresholds are proportional to the pedestal and this dependency might be very different for visual and vestibular cues, which in turn may result in pedestal dependent differing cue reliabilities.

### Differences between heading- and displacement discrimination

Recent literature shows that during heading perception one receives accumulating evidence for direction of travel (i.e., leftward or rightward) during self-motion and hence heading estimates’ certainty will increase over time [19]. For displacement discrimination, however, it is unlikely that evidence will increase over time. In order to estimate the travelled distance one needs to take the double integral of the acceleration profile of the entire movement. Furthermore, in a comparison between heading-discrimination- and a displacement-discrimination task it was shown that heading discrimination dependent on movement directions relative to the head, but not movement directions in world coordinates. In contrast, displacement discrimination depended on movements in world coordinates, but not movements relative to the head [9]. Therefore, the processing of sensory information for displacement discrimination during self-motion might be very different. Up to a certain level, our results are in line with the findings of reliability based weighting during heading estimation. However, during heading estimation a vestibular overweighting as been reported consistently [2, 10, 11], whereas we did not observe such an overweighting with respect to optimal integration schemes. How these differences are manifested at the neuronal level is unknown. It is likely that sets of neurons can be found that specifically code for displacement as there are neurons specifically tuned to coding heading angles [3, 17, 32].

In most experiments regarding relative cue weighting in self-motion perception there is an inherent concern that the visual only condition introduces a sensory conflict between the visual and vestibular information. That is, while observing optic flow, the visual system detects movement while the vestibular system specifies a stationary position. Consequently, the visual only condition might not be a purely unisensory condition. We expect this influence to be minor in our task, since [16] showed that in non-human primates there was no difference in visual heading estimation between labyrinthectomized- and control animals.

The results on the thresholds show that there is a significant reduction of the combined threshold with respect to the lowest single cue thresholds, but only for the 100% and 60% visual motion coherence conditions. This is in line with the theory on optimal integration in which the reduction of the combined cue threshold is strongest when the two single cues have equal thresholds. An increasing difference between the single cue thresholds, as is the case in the 20% coherence condition, will henceforth result in only a small reduction of the combined threshold with respect to the lowest single cue threshold.

Finally, our data show that adding discrepancies between the single cues increases the combined thresholds in the 100% and 60% visual motion conditions compared to the combined thresholds in congruent conditions. This is consistent with results on heading perception, showing that adding discrepancies affects the integration process of visual and vestibular cues, also when subject were not aware of any discrepancies [11].

## Conclusion

In this study, we showed that the integration of visual and vestibular cues during displacement estimation is in line with two predictions of probabilistic inference theory. We showed that perceptual precision is increased when both cues are combined with respect to either of the single cues and that cue weighting is dynamically based on relative cue reliability.
